# Cost comparison of in-person and telehealth modalities for a suicide safety planning group intervention: interim results from the “Project Life Force” randomized clinical trial

**DOI:** 10.3389/fpsyt.2023.1215247

**Published:** 2023-10-17

**Authors:** Rebecca A. Raciborski, Kyra K. Hamerling-Potts, Emily L. Mitchell, Sarah R. Sullivan, Nidhi Kapil-Pair, Sara J. Landes, Shari Jager-Hyman, Marianne Goodman

**Affiliations:** ^1^VA Center for Mental Healthcare and Outcomes Research, Central Arkansas Veterans Healthcare System, North Little Rock, AR, United States; ^2^Behavioral Health Quality Enhancement Research Initiative (QUERI), Central Arkansas Veterans Healthcare System, North Little Rock, AR, United States; ^3^Evidence, Policy, and Implementation Center, Central Arkansas Veterans Healthcare System, North Little Rock, AR, United States; ^4^VISN 2 Mental Illness, Research, Education, Clinical Center (MIRECC), James J. Peters Veteran Affairs Medical Center, Bronx, NY, United States; ^5^Department of Psychology, The Catholic University of America, Washington, DC, United States; ^6^Department of Psychiatry, Vagelos College of Physicians and Surgeons, Columbia University Medical Center, New York, NY, United States; ^7^Department of Psychiatry, University of Arkansas for Medical Sciences, Little Rock, AR, United States; ^8^Department of Psychiatry, University of Pennsylvania, Philadelphia, PA, United States; ^9^Department of Psychiatry, Icahn School of Medicine at Mount Sinai, New York, NY, United States

**Keywords:** cost analysis, cost minimization, telehealth, suicide prevention, safety planning intervention, group intervention, veterans, randomized clinical trial

## Abstract

Suicide prevention is a clinical priority for the US Veterans Health Administration. Evidence-based interventions, including developing a suicide safety plan, are recommended practices and are becoming more widespread. Adaptations to further augment safety planning include a manualized group intervention (Project Life Force, PLF) that combines safety planning with the teaching of skills to maximize use of the plan. A multi-year randomized controlled trial to test efficacy of PLF compared to treatment as usual is currently in progress. However, approximately a year into the study, in-person groups were converted to telehealth groups due to the COVID-19 pandemic. This study compares the per-veteran cost of PLF when delivered in-person versus by telehealth using preliminary trial data from the first 2.5 years of the trial. Cost to deliver PLF was obtained from the Veterans Health Administration’s Managerial Cost Accounting data, which relies on activity-based costing. We found no significant differences in the average number of sessions or average group size between in-person and telehealth. However, the cost per group session was lower for the telehealth modality and this led to significant overall per-veteran savings. While efficacy data comparing from the two arms is still underway and we await the ongoing RCT results, our interim cost analysis highlights potential savings with the telehealth modality.

## Introduction

Suicide is the 13th leading cause of death in the United States with a suicide death every 11 min (1). However, veterans are disproportionally affected by suicide; from 2001 through 2020, age- and sex-adjusted suicide rates of veterans exceeded those of non-veteran adults (2). The 2020 veteran suicide rate was 57.3% higher than that of civilians (2). This prompted the Veterans Health Administration (VHA), the health system of the US Department of Veterans Affairs (VA), to identify suicide prevention as its number one clinical priority and to support research focused on interventions that target high-risk populations. One such intervention is “Project Life Force” (PLF), a manualized suicide safety planning group intervention (3, 4).

Safety Planning Intervention (SPI) has been designated a “best practice” by The Suicide Prevention Resource Center (5), which means that the intervention meets programmatic guidelines and standards of accuracy and safety. SPI is strongly recommended by an array of governmental and not-for-profit agencies in the US and Canada, and it is included in the joint VA and DoD Clinical Practice Guidelines (6). A core component of SPI is construction of a suicide safety plan (SSP). The SSP is a prioritized, sequential written list of coping strategies and sources of support developed collaboratively by patient and clinician to mitigate suicide risk for patients with suicidal ideation, and suicidal behavior (7, 8). The SSP instructs one to recognize personal warning signs of suicide; use internal coping strategies; engage social contacts that can offer support and serve as distraction from suicidal thoughts; contact family members or friends who may help resolve a crisis; provide contact information for VA professionals; and specify steps for how to make the immediate environment safer (9, 10).

Suicide safety planning is one of the few empirically based suicide-specific interventions (7, 11). A recent systematic review of suicide safety planning identified 26 articles worldwide (8) and noted safety planning to be a valuable indicated intervention for suicide-related distress. Quantitative findings included improvement in suicidal ideation and behavior, depression, hopelessness, and reductions in hospitalization. Moreover, a meta-analysis of safety planning compared to a control condition (*n* = 6) found significantly improved outcomes for suicidal behavior (12). There is growing interest in delivering interventions in groups given the benefits of peer support (13). While there has been some limited use of group therapy with a suicide focus (13, 14) and group therapy where safety planning occurs (11), to our knowledge, PLF is the first manualized group intervention focused on development of an SSP.

A trial to compare the efficacy of PLF adjunctive to usual care versus usual care alone is currently underway (4); PLF has been provided in-person and via telehealth in the trial. PLF was conceived of as an in-person intervention, but it transitioned to a telehealth format as part of a larger rapid expansion of telemental health as VHA responded to the COVID-19 pandemic (15). Although pandemic protocols have ended, the higher rate of telemental health use is likely to be sustained (16), especially within VHA given its pre-pandemic momentum for telehealth (17).

The combination of group formats with telehealth delivery may be especially compelling. There is growing interest in group formats given the benefits of peer support. Group formats may also be more cost-effective than individual treatment (18–20). Similarly, there is some evidence that telehealth is cost-effective (21, 22) and even may have lower cost than in-person visits (23), but a favorable comparison has not been found by all authors (24). Yet, little is known about the potential cost differences of delivering group interventions via telehealth versus in-person. This is policy-relevant information for VHA and similar publicly funded healthcare providers. As a taxpayer funded institution, VHA is obliged to be a good steward of government resources while providing the best care to veterans. Identifying effective treatment alternatives for suicide prevention that can be provided while simultaneously minimizing expenditures to conserve funds for use elsewhere is key to successfully meeting this obligation. Using interim trial data, we sought to determine if there was a difference in per-veteran treatment cost to VHA between in-person and telehealth PLF and to examine reasons for potential differences in cost.

## Method

The presented analysis uses interim results from a single site within a multi-site, multi-year trial (described below) to investigate cost differences between in-person PLF and telehealth PLF (PLF-T). This study uses cost-minimization analysis to examine differences between the two modalities used to deliver PLF. Cost-minimization analysis is conducted under the assumption that treatment options are equally effective and focuses on the relative differences in cost to obtain desired outcomes. In this case, the treatment options being compared are PLF and PLF-T. The primary clinical outcome of the trial is time to suicidal behavior and secondary outcomes include hopelessness, suicidal ideation, and suicide-related coping. A pilot established that PLF is safe and is likely to be efficacious (3). While no data exists about equivalence in outcomes between PLF and PLF-T, the literature on the use of telehealth to provide mental health care has consistently shown that telemental health is as effective as in-person care (25, 26).

### Trial design

The Group (“Project Life Force”) vs. Individual Suicide Safety Planning trial is a multi-site, four-and-half-year randomized controlled trial (RCT) is designed to establish the efficacy of PLF adjunctive to usual care compared to usual care alone (4). VHA mandates that all individuals who are suicidal and discharged from inpatient care and individuals on the suicide high-risk list receive monitoring, outreach, and involvement of a suicide prevention coordinator and clinical management that constitutes standard VHA care for suicidal individuals. Usual care also includes development of a safety plan.

PLF adapts safety plan development to a group format in which veterans develop and enhance individually tailored safety plans over 10 sessions. The intervention also integrates skill-based and psychoeducational approaches, such as teaching distress tolerance, emotion regulation, and interpersonal skills, to optimize the effectiveness of veterans’ safety plans. Additional sessions include lethal means safety, augmenting physical well-being, strategies for sharing their plan with family or significant others, and how to access crisis line services. Importantly, the group format aims to mitigate loneliness and foster increased “belongingness” (27), both key risk factors for suicide. The group cohort model facilitates connection among veterans and aims to build a sense of community and social net, which is essential for those who are otherwise lonely and isolated.

Veterans are eligible to participate in the PLF trial if they are age 18 years or older; have an outpatient encounter for suicidal ideation or attempt, have been discharged from an inpatient unit for suicidal ideation or attempt, or have been placed on the high-risk suicide list maintained by suicide prevention coordinators; and have a provider who was willing to coordinate with study staff. Providers referred potential subjects with suicidal ideation or attempt from outpatient encounters or after inpatient discharge and eligibility was confirmed by a Columbia Suicide Severity Rating Scale (28) of 3 or greater.

Veterans are excluded from the PLF trial if they are unable to provide informed consent; are unable to speak English; are unable to supply a verified emergency contact; or are unable to attend outpatient group sessions or tolerate the group format. They are also excluded if they have cognitive difficulties that impair consent, have substance use dependence that requires medically supervised withdrawal, have schizophrenia, or are participating in another interventional RCT.

An objective fidelity scale was developed to assess core features of the PLF structure, contents, and treatment principles along with general clinical competence. Fidelity measures are obtained for a random sample of approximately 20 percent of sessions. Using a 6-point Likert scale (0 = unacceptable and 5 = excellent) to demonstrate adequate adherence to the intervention, clinicians were required to maintain an average score of 4 or higher. While the study requires additional supervision and adherence monitoring if a clinician’s average rating falls below 4, fidelity scores throughout the trial have exceeded this threshold. In addition, safety plan quality is being graded for PLF and individual safety planning with an exploratory aim to examine quality differences. Goodman and colleagues (4) describe additional details of the trial.

The first PLF session occurred on December 19, 2018. We planned for all PLF sessions to be in-person group meetings. In March 2020 however, due to the COVID-19 pandemic, VHA closed facilities for most in-person operations. To maintain access for participants, we quickly developed strategies to conduct all study procedures virtually, including consenting of patients and assessment. In-person group visits also were transitioned to telehealth via video conferencing services in March 2020. In-person group visits have not resumed at study sites. This unplanned transition effectively created an additional arm for the trial (PLF-T) because all veterans randomized to receive the group intervention after the transition to telehealth received only PLF-T.

### Study sample

**Figure 1:**
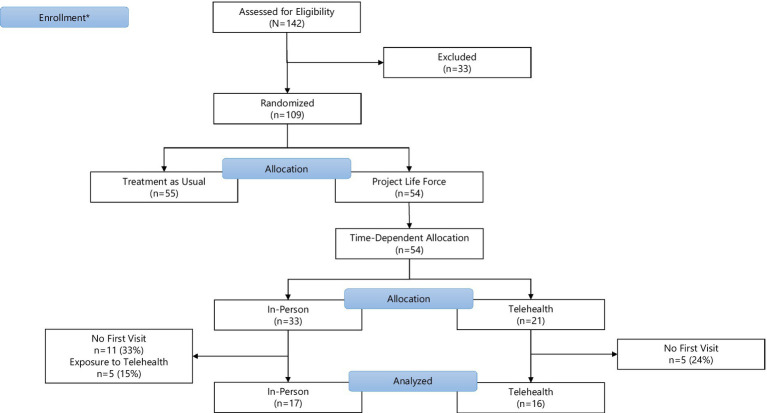
CONSORT flowchart. *Enrollment for this analysis restricted to the first 2.5 years of the 4-year long PLF trial.

We use interim data collected on subjects randomized to the PLF intervention at the primary study site (Bronx, NY) prior to June 30, 2021. Of the 54 veterans who qualified, 33 veterans (61%) were randomized prior to March 1, 2020, comprising the “in-person” PLF cohort. The 21 veterans (39%) randomized on or after March 1, 2020 comprise the PLF-T cohort. Within the in-person PLF cohort, five subjects (15% of the in-person cohort) were exposed to both modalities because they began in-person PLF and remained active after pivoting to PLF-T. These subjects are excluded from this analysis. An additional 16 subjects across both cohorts (30%) randomized to the intervention were excluded from the analysis because they did not attend any PLF sessions. The final sample size of 33 is composed of 17 veterans in the in-person PLF cohort (52%) and 16 veterans in the PLF-T cohort (48%). Figure 1 presents the CONSORT flowchart.

### Measures

Visits occurring as part of the PLF trial were tracked by study personnel using clinical notes in each veteran’s electronic health record (EHR). For the purposes of this analysis, we only include the 10 visits that comprise the core of PLF. We link these visits to data from VHA’s Managerial Cost Accounting Office (MCAO) to obtain the cost of each visit. These data are stored in the VHA Corporate Data Warehouse along with the EHR data.

MCAO uses activity-based accounting to assign a cost to different encounter types based on the labor required to treat the patient. The MCA data also includes variable costs for supplies consumed during the visit (e.g., printouts given to patients), direct overhead charges (e.g., shared staff support), and indirect overhead charges (e.g., building space). All costs are in US dollars and are actual accounting costs at the time of measurement.

### Analytic plan

PLF and PLF-T share many of the same key features. For this analysis, the relevant features of the intervention are that the treatment course is expected to last for a minimum of ten 90-min sessions; each session is run by two therapists; and, due to rolling admission, veterans are not required to complete sessions sequentially. Veterans could attend up to 4 “booster” sessions beyond ten, but they were not required to. PLF-T differed in staffing because the team added a third non-clinical staff member at all sessions to provide real-time technical support for video conferencing. Additionally, after the transition to PLF-T, the lead therapist from the second study site became the co-facilitator for the virtual groups. Previously, the co-facilitator for the groups was a post-doctoral fellow. Staffing the co-facilitator role with a psychologist is not considered a core feature of the PLF-T intervention.

Because our interest is in the difference in cost between in-person PLF and PLF-T, we focus our analyses on those expenses that vary between the two modalities of delivering PLF. We make the simplifying assumption that “treatment as usual” costs are not related to PLF modality and exclude them from the cost calculation in this early stage. Other costs that are the same regardless of PLF treatment modality include the cost of printing handouts and a required one-time eight-hour training for clinicians before administering PLF. Mailing costs are only incurred for PLF-T. However, separate mail charges were not tracked as part of the trial because patient mailings within VHA commonly are provided by the facility’s mail service. Optional booster visits are excluded because there was no time constraint placed on their completion, making it difficult to know how many each veteran would ultimately use.

For this analysis, we also exclude the cost of training providers to use telehealth technologies. VHA has more than a decade of using telehealth to provide mental health services (29), so providers already had familiarity with the systems. Given VHA’s emphasis on maintaining veterans’ access to care for participants during pandemic-related closures, all VA providers had mandatory training on using the Veteran Video Conferencing system. The training lasted one hour and was provided virtually. Providers also had access to an additional 8 h best-practices course and a VHA-wide community of practice to provide additional support.

We computed cost given the observed average number of sessions completed and average group size for each of the two modalities. We used *t*-tests to compare means by modality. Because most veterans who completed all 10 sessions took longer than 10 weeks to do so, we had planned to adjust the average number of sessions completed in the virtual group to avoid artificially biasing the mean in this group down. However, in the absence of any difference in number of sessions between the two modalities, we omitted this planned step. We conducted all statistical analyses using Stata version 17.0 (StataCorp, College Station, TX; RRID:SCR_012763).

## Results

Subjects in the analytic sample made a combined 210 visits during the study window across both modalities; 111 visits were made by subjects in the in-person PLF cohort and 99 were made by subjects in the PLF-T cohort. We were unable to match 7 visits (3.3%) to the MCA cost data. Although correspondence with MCAO indicated that the variable direct (labor) cost of group visits would change with the number of group members in attendance, we ultimately found this not to be the case. Instead, costs per unit (single visit of standard visit duration) were constant within fiscal year. The cost of each unit for visits in different time periods is shown in Table 1.

**Table 1:** Unit cost for PLF encounters by type.

	Arm	
	PLF	PLF-T
Time period		
FY19	$239.78	
FY20	$316.22	$161.55
FY21		$149.38
All (average unit cost)	$251.41	$154.34

Additionally, when reviewing the cost data, we found that 36 PLF-T visits (38%) were incorrectly coded as the higher cost in-person modality. For these visits, we substituted a matched telehealth visit cost and applied any adjustment for volume of service (e.g., if the record indicated 2 units, we multiplied the unit cost of PLF-T by 2 to obtain the total visit cost for that subject). We did not fill in data for subjects with missing cost records because their volume measurements were also missing. There were no differences in the proportion of subjects with missing cost data between the PLF and PLF-T arms (*z*-statistic for test of proportions = −0.54; data not shown).

Overall, veterans assigned to the PLF arm completed an average 6.4 sessions (95% CI: 4.9 to 7.8) in groups averaging 2.3 veterans per session (95% CI: 2.1 to 2.6). There were no significant differences in average number of sessions (difference = 0.34; *t* = 0.25) or average group size (difference = 0.16; *t* = 0.72) between in-person PLF and PLF-T. Among the 16 veterans who completed all 10 PLF sessions, the average length of time to completion was 102 days after attending the first session (95% CI: 83 to 122). This is significantly higher than the expected 63 days if sessions are attended weekly with no gaps (*t* = 4.2, *p* < 0.001).

On average, in-person PLF costs about $264 per session (SD = $68.03). By comparison, PLF-T costs only about $158 per session (SD = $35.48). The difference in mean cost, $105.33, is statistically significant at the 95% confidence level (*t* = 13.5, CI: $90.00 to $120.65). The expected in-person PLF per-veteran cost is $1,722.01, holding number of sessions and group size constant; PLF-T costs about $690 less at an estimated $1,031.66 per veteran. These results are presented under the “Average” row under “Cost per subject” in Table 2. As part of a sensitivity analysis, we computed costs for veterans completing 7 (core program) and 10 (full program) sessions. In this case, we anticipate that the per-person cost for in-person PLF would be about $746 higher than PLF-T for the core program and $1,118 higher than PLF-T for the full program. These results are shown in the last rows of Table 2.

**Table 2:** Estimated PLF attendance and cost by modality.

	PLF	PLF-T	Difference (95% CI)	*t* or *F* statistic
Number of subjects	17	16		
Number of sessions	111	99		
Average sessions/subject^a^	6.5	6.2	0.34 (−2.50; 3.19)	0.25
Average group size^a^	2.4	2.3	0.16 (−0.29; 0.61)	0.72
Cost per subject^b^				
Average	$1,722.01	$1,031.66	$690.35 (554.11; 826.58)	108.11
Estimated 7 sessions	$1,844.98	$1,098.71	$746.27 ($609.29 $883.25)	124.96
Estimated 10 sessions	$2,661.84	$1,544.06	$1,117.78 ($934.24; $1,301.32)	156.15

a Test statistic is for a t test of the null hypothesis of no difference in groups.

b Test statistic is for an *F* test of the null hypothesis of cost difference greater than $0.

## Discussion

We found that PLF-T offers per-veteran savings of about $690 over in-person PLF, an encouraging finding as telehealth becomes more widespread. These savings come from the lower per-visit cost of PLF-T rather than less intensive use of sessions by veterans. We found no differences in attendance between the two modalities. The group cohort model is intended to facilitate connection among veterans and aims to build a sense of community and social net, which is essential for those who are otherwise lonely and isolated. On the other hand, the virtual modality could have been less supportive, leading veterans to attend fewer groups. It is reassuring that we found no evidence of lower attendance in the virtual groups, suggesting that PLF-T may prove to be as efficacious as PLF. If ultimately shown to be efficacious and cost-efficient at the end of the trial, PLF-T could be integrated into VA’s “gold standard of care”. Regardless of modality, per-session intervention costs would be lower if group sizes had hit the planned target of five veterans per session. Therefore, future replications of PLF in additional sites should include implementation strategies designed to increase veteran attendance.

### PLF costs and effect on VHA budgets

Our findings that PLF-T is less costly than PLF is in line with a broader literature that has generally found that telemental health is more cost-effective than providing in-person care (22). Cost depends on perspective though; some authors have found that telehealth is more expensive when start-up costs are included (24). Health systems that are not able to leverage an existing telehealth infrastructure may find that PLF-T would be more costly. Additionally, because this analysis was conducted from the perspective of VHA using cost incurred during the trial our estimates do not include the cost of providing video conferencing equipment to participants; although VHA will provide equipment to veterans, if necessary, this was not required for any of the trial participants. Others have found that inclusion of patient technology expenses can alter conclusions about cost differences between telehealth and in-person care (24). Use of PLF-T in veterans with fewer digital resources could result in less favorable comparisons.

Healthcare budgets within VHA are generally locally controlled, with individual facilities having the flexibility to choose which interventions to implement as they pursue national mandated performance targets and policy goals. The cost-minimization analysis provides an indication of how much cost would need to be offset by changes in medical care use elsewhere. RCT outcome data on mental health care service use is not yet available but the possibility of PLF minimizing use of costly services such as emergency mental health care or hospitalizations for suicide attempts may yield sufficient savings to offset the program cost. More importantly, the results here suggest that PLF may ultimately also prove cost-effective. With a total cost of around $2,660 for the full 10 sessions per veteran using the more expensive in-person modality, it is possible that sufficient benefits accrue to offset these costs. At the conclusion of this trial, the study team plans to compare differences in emergency room visits, inpatient hospitalizations, and outpatient mental health visits. A formal budget impact analysis at the conclusion of the study may elucidate the net effect of PLF on an individual VHA facility’s budget.

### PLF efficacy

A pilot study suggests that PLF may be efficacious (3), although this is not yet established; its efficacy relative to individual safety planning is currently being assessed in the current RCT. Additionally, there is not yet evidence to determine if in-person group sessions have better outcomes than virtual group sessions. The data from this analysis include subjects engaged in the treatment during the height of the COVID-19 pandemic (March 2020 to June 2021) and when the transition to virtual care was in its early stages. Other data from our ongoing RCT suggests that later PLF telehealth participants (from July 2021 to September 2022) may have increased attendance to about 8 sessions on average per veteran, inclusive of those who have not yet had time to complete all 10 sessions. This suggests PLF-T attendance may be superior to in-person PLF sessions, which is consistent with what others have found about the telehealth modality more generally (30, 31).

### Limitations

This study is limited by its restriction to a single site. Facility-specific costs can vary widely by region and may especially limit the generalizability of findings with respect to the cost of in-person care. Further, the current estimates present only the difference between in-person PLF and PLF-T, not the actual cost of the PLF intervention because common costs are excluded. In particular, the cost of training clinicians to provide PLF, required regardless of modality, and the cost of training clinicians to use telehealth technologies for PLF-T are not included in the cost estimates presented here. However, the effect of the training cost on per-veteran cost decreases in importance over time as each provider treats more patients.

Our conclusions about cost may be further limited by our decision to use the staffing model employed within the trial. Specifically, a senior research psychiatrist was paired with an advanced psychology fellow to conduct in-person PLF visits and paired with a senior psychologist during virtual PLF visits. Neither staffing model is likely to be used with wider adoption. We consulted with VHA operational partners in the office of Mental Health and Suicide Prevention about how such visits would be staffed with broader adoption. The consensus view was that a psychologist would be paired with either a trainee (post-doctoral psychologist) or another mental health provider such as a licensed clinical social worker; both staffing models would have lower cost. Finally, we computed cost using the cost of visits as currently valued in VHA’s MCA data with standard accounting codes. Several PLF visits in the beginning of transition to telehealth appear to have been incorrectly coded with in-person visit accounting codes. We made a simple substitution of a standard telehealth visit cost for visits that occurred after the transition. Subsequent analyses with a larger sample can use methods to statistically adjust for coding errors (e.g., imputation of a corrected cost).

### Next steps

At the conclusion of the RCT, a revised cost analysis will incorporate data from the other PLF sites, which did not have veterans receiving PLF-T at the time this interim cost study was approved. Cost analyses could be further enhanced by accounting for changes in health status. Depression is an extremely debilitating disease; the social value of these changes could be incorporated in future analyses. The PLF RCT is examining key veteran health-related outcomes, including suicide and quality of life outcomes like the Beck Depression Inventory. Similar quality of life benefits may occur for family members and caregivers. The quality-of-life benefits are balanced against costs that are incurred directly or indirectly by veterans and their families. The appropriate framework to incorporate these benefits and costs is a cost-effectiveness analysis. While collecting quality of life measures is beyond the scope of the current trial that this supplement was funded under, future expansion of PLF in a larger trial may consider adding data collection necessary to support a cost-effectiveness analysis.

## Data availability statement

The datasets presented in this article are not readily available because, under U.S. regulations, the Department of Veterans Affairs retains ownership of the dataset and the authors may not grant permissions to access. However, the informed consent form permits a subset of deidentified primary data collected for the trial to be shared for qualitative analyses only. Requests to access deidentified primary trial data should be directed to MG, marianne.goodman@va.gov.

## Ethics statement

The studies involving humans were approved by James J. Peters Veterans Administration Veterans Center Institutional Review Board. The studies were conducted in accordance with the local legislation and institutional requirements. The participants provided their written informed consent to participate in this study.

## Author contributions

RR and MG contributed to conception and design of the study. SS developed the patient data collection and tracking protocols. EM, KH-P, and SS collected and organized patient clinical records. EM and KH-P performed quality checks on patient clinical records data. KH-P and RR performed descriptive analyses. RR performed the economic analysis. RR and MG wrote the first draft of the manuscript. RR, MG, NK-P, SL, and SJ-H interpreted the results and contributed to discussion of their clinical and policy contextualization. All authors contributed to the article and approved the submitted version.
